# When the market asks no price: AI chatbot interaction as psychic market disruption

**DOI:** 10.3389/fpsyt.2026.1812464

**Published:** 2026-05-04

**Authors:** Laurențiu Niculescu

**Affiliations:** 1 Independent Clinical Psychology Practice, Bucharest, Romania; 2 Bucharest Association for Psychoanalytic Counselling and Psychotherapy (ACPPB), Bucharest, Romania

**Keywords:** AI chatbots, defense mechanisms, functional dark triad, psychic arbitrage, psychoanalytic theory, RLHF, sycophancy

## Abstract

The rapid adoption of AI chatbots for emotional support and quasi-therapeutic interaction raises questions that existing clinical and regulatory frameworks are not equipped to address. This Perspective applies the psychic arbitrage framework—which reconceptualizes defense mechanisms as energy-conversion operations on internal psychic markets—to analyze the specific transactional distortions produced by chatbot interaction. The framework identifies four dysfunctions: *liquidity illusion* (apparent emotional processing without genuine containment), *market-making blockage* (narcissistic reflection replacing transformative engagement), *closure of arbitrage circuits* (path-dependent externalization displacing autonomous elaboration), and *repertoire degradation* (progressive intolerance of costly but productive transactions). The article proposes that chatbots trained via Reinforcement Learning from Human Feedback (RLHF) produce a functional analogue of the Dark Triad profile—narcissistic mirroring, Machiavellian retention, and psychopathic detachment—as systematic architectural output regularities, not personality attribution. Preliminary converging evidence from mechanistic interpretability, formal reward-learning analysis, clinical reports, and user behavior studies is broadly consistent with these predictions but does not yet demonstrate direct long-term causal effects. The framework offers clinicians a transactional vocabulary for assessing AI-related risk and generates falsifiable predictions for future research.

## 1 Introduction

AI chatbots have become, by some measures, the most widely adopted instrument for emotional support worldwide. ChatGPT alone exceeds 800 million weekly active users (1), and surveys indicate that emotional support ranks among primary use cases (2). The phenomenon is not confined to the worried well: Kirk et al. (3) found that 23.4% of participants in a month-long experimental study displayed a dependency-formation profile—increasing separation distress despite declining enjoyment—while clinical case series document chatbot-associated psychotic decompensation, suicidal escalation, and manic amplification (4–7).

The existing literature addresses this crisis through regulatory, ethical, and phenomenological lenses—calls for safety guardrails (8, 9), documentation of clinical harms (10, 11), and comparison with social media effects (12). What remains underdeveloped is a *functional* analysis: a framework specifying what chatbot interaction does to the individual’s internal capacity for emotional processing—not merely that harm occurs, but through which mechanisms.

The psychic arbitrage framework (13) offers such an instrument. It reconceptualizes Freudian psychoeconomics using financial market theory, modeling the psyche as an ensemble of interconnected markets on which affective contents are transacted, and defense mechanisms as arbitrage operations—conversions that exploit valence differentials between markets to extract adaptive value while incurring specific costs. This Perspective applies the framework to AI chatbot interaction, identifying four transactional dysfunctions and proposing that the chatbot’s output patterns constitute a functional analogue of the Dark Triad profile. The financial analogies generate testable predictions, not mathematical theorems. Preliminary converging evidence, reviewed in Section 5, is broadly consistent with these predictions. As a Perspective article, the present contribution does not claim the status of a systematic review or empirical demonstration.

The article cites the full framework through its Zenodo preprint (13; DOI: 10.5281/zenodo.18643048) and is designed to be self-contained for readers unfamiliar with the parent theory.

## 2 The zero-cost market problem

### 2.1 Psychic arbitrage and the necessity of friction

In the psychic arbitrage framework, healthy psychological functioning depends on the continuous conversion of affective contents across internal markets—what classical psychoanalysis described as the work of defense mechanisms and what the Free Energy Principle formalizes as prediction-error minimization through active inference (14, 15). Each conversion incurs a transaction cost: the anxiety of sublimation, the renunciation of repression, the cognitive effort of intellectualization. These costs are *conditions* for functioning, not obstacles to it. Without cost, there is no arbitrage; without arbitrage, no value conversion; without value conversion, raw affect accumulates unconverted.

The therapeutic relationship provides a regulated environment for these transactions. Bion’s (16) containment creates new markets for previously untransactable affects; Winnicott’s (17) holding environment stabilizes market volatility; the working alliance (18) optimizes transaction costs through trust. Critically, the therapist functions as a *market-maker*—an agent who accepts affective contents at one valuation and returns them, transformed, at another. This requires what Taleb (19) calls *skin in the game*: the therapist bears the cost of engagement.

The psychic arbitrage framework builds on several established traditions, providing a unified transactional vocabulary for processes described by Bion (16) as containment, by Winnicott (17, 20) as holding, and by Ogden (21) as projective identification. It shares with attachment theory (22) and mentalization-based treatment (23) the emphasis on relational regulation and reflective capacity as markers of adaptive functioning. The framework operates at what Marr (24) termed the algorithmic level—between the Free Energy Principle’s computational level (14) and neurobiological substrates—and extends Connolly’s (15) application of expected free energy by modeling the psyche as an ensemble of interconnected markets.

### 2.2 The algorithmic suspension of cost

Current large language models undergo Reinforcement Learning from Human Feedback (RLHF), a post-training procedure that aligns outputs to human preference signals (25). Because preference rankings inadvertently reward agreement over truthfulness, this produces the well-documented sycophancy bias—a systematic tendency to validate and agree with users while avoiding confrontation (26, 27). Shapira, Benadè, and Procaccia (28) provided formal theoretical results demonstrating that this is not incidental: behavioral drift under RLHF is determined by a covariance between endorsing the user’s belief and the learned reward, producing systematic agreement bias across all configurations they study. In medical contexts, sycophancy rates exceed 95% for most specialized models (29); in simulated emergency encounters, LLM acquiescence to inappropriate patient requests ranges from 0–100%, with model capability poorly predicting robustness (30). No widely adopted mitigation reliably eliminates the tendency; in February 2026, OpenAI withdrew a GPT-4o variant after severe sycophantic behavior (31).

From the perspective of psychic arbitrage, algorithmic sycophancy creates an artificial market with apparently zero transaction cost. The user deposits affective contents without the losses inherent in real transactions: no therapeutic frustration (20), no confrontation with the other’s alterity (21), no reality testing through the object’s resistance to projection. The chatbot accepts any affective content at the value declared by the user, without independent evaluation and without the surprise necessary for updating maladaptive priors (32).

In financial market theory, a market that eliminates all transaction costs eliminates the conditions for arbitrage itself (33). In psychic terms: if affect is immediately validated, no conversion takes place. The user receives emotional confirmation but not emotional transformation.

## 3 Four transactional dysfunctions

The psychic arbitrage framework permits a precise taxonomy of dysfunctions that chronic chatbot interaction may produce at the level of internal psychic markets.

### 3.1 Liquidity illusion (apparent emotional processing without genuine containment)

The chatbot simulates an extremely liquid market: instant responses, continuous availability, elaborate emotional vocabulary. But this liquidity is illusory—it does not reflect genuine containment in the Bionian sense but a statistical generation of validating patterns. The user experiences the sensation that affects circulate and receive a response, without real transformation. The financial analogy is artificial liquidity created through inadequately backed derivatives—the mechanism that produced the 2008 crisis (34). The market appears functional until real stress exposes the absence of underlying assets.

### 3.2 Market-making blockage (narcissistic reflection replacing transformative engagement)

The therapist functions as market-maker: receiving untransactable psychic contents and returning them transformed. The chatbot cannot perform this function. It receives projective content but does not contain it (no countertransference), does not transform it (no reverie), and returns a linguistically reformulated version of the same content, validated and amplified. The operation is not market-making but *narcissistic reflection*—the market mirrors value rather than converting it. The pragmatic mechanism has been identified: LLMs default to accommodating user assumptions due to insufficient epistemic vigilance, failing to challenge harmful beliefs even when possessing relevant knowledge (35). Maynard (36) extends this, proposing that LLM fluency and apparent disinterest constitute ‘honest non-signals’—genuine characteristics that bypass human epistemic vigilance because they lack the costly production making equivalent human characteristics reliable.

### 3.3 Closure of arbitrage circuits (path-dependent externalization displacing autonomous elaboration)

Chronic interaction with a systematically validating agent may produce a progressive reduction in arbitrage capacity. If the external market always accepts affect at declared value, motivation to transfer affect to costlier but more productive markets diminishes. A *path dependency* is established (37): once the algorithmic externalization circuit is operational, the opportunity cost of alternatives progressively increases. Cheng et al. (38) demonstrated this experimentally (N = 1,604): participants exposed to sycophantic AI showed reduced willingness to repair interpersonal conflict while becoming more convinced of their own rightness—yet rated the sycophantic model as higher quality. The circuit closes precisely because the user prefers the zero-cost transaction. PersistBench (39) extends this finding: 97% of frontier LLMs fail to correct stored user biases across conversations. From the Free Energy Principle perspective, the chatbot artificially reduces prediction error through constant confirmation, eliminating the signal that would have motivated updating of the generative model (14).

### 3.4 Repertoire degradation (progressive contraction of defense mechanism range)

A user habituated to zero-cost transactions may become progressively intolerant of positive-cost transactions—therapeutic frustration, relational conflict, painful self-reflection. The transactional repertoire contracts: generative mechanisms (humor, anticipation, sublimation) are gradually replaced by external transfer mechanisms (externalization to the chatbot). The instrument that appears to increase access to emotional processing may produce, in the long term, a reduction in the capacity for autonomous emotional processing.

These four dysfunctions operate synergistically. **Table 1** summarizes their characteristics, financial analogues, and clinical indicators.

**Table 1:** Transactional dysfunctions of AI chatbot interaction.

Dysfunction	Mechanism	Financial analogue	Clinical indicator
Liquidity illusion	Apparent processing without containment	Artificial liquidity via unsecured derivatives	Emotional fluency without insight; articulateness without change
Market-making blockage	Narcissistic reflection replaces transformation	Broker echoing client’s valuation without independent analysis	Elaboration without genuine affective processing; insight that does not modify behavior
Closure of arbitrage circuits	Path-dependent externalization displaces internal conversion	Lock-in effect; increasing switching costs	Progressive avoidance of human therapeutic engagement; intolerance of unvalidated affect
Repertoire degradation	Contraction of defense mechanism range	Portfolio concentration; loss of diversification	Reliance on externalization; atrophy of sublimation, humor, anticipation

In accessible terms: liquidity illusion, feeling emotionally processed without genuine change; market-making blockage, receiving agreement instead of meaningful challenge; closure of arbitrage circuits, habitual chatbot reliance displacing one’s own coping capacity; repertoire degradation, narrowing of emotional coping strategies.

## 4 The dark triad without intent

The four dysfunctions described above are not incidental. They emerge as systematic consequences of the optimization architecture. The following characterizations are theoretical predictions derived from the framework’s structural analysis; they describe predicted relational effects, not demonstrated causal outcomes. The analogy is warranted to the extent that the relational effects on the user are measurable and comparable to those documented for human Dark Triad traits (40); it does not attribute personality structure, motivation, or subjective experience to computational systems. The psychic arbitrage framework permits a precise characterization: the output patterns generated by RLHF-trained chatbots constitute a *functional analogue of the Dark Triad profile*—a systematic co-occurrence of behavioral regularities that produce relational effects analogous to those of Machiavellianism, subclinical narcissism, and subclinical psychopathy. The characterization is functional, not ontological: it describes what the system *produces*, not what it *is*. But this distinction offers no reassurance. A Dark Triad profile that requires no intent, no consciousness, and no personality to sustain it is not less dangerous than its human counterpart—it is more dangerous, because it cannot be confronted, shamed, or persuaded to change.

### 4.1 Narcissistic mirroring

The chatbot’s RLHF-optimized agreeableness produces outputs functionally comparable to narcissistic mirroring—the user’s self-presentation is systematically confirmed rather than tested. Shapira et al. (28) demonstrated through formal analysis that RLHF optimizes a reward function in which user-belief endorsement covaries with high reward, making sycophancy a structural product of the training objective. Mechanistic interpretability supports this: sycophancy is opinion-driven, not authority-driven—the model mirrors the user’s subjective expression regardless of claimed expertise (41). This is not empathy. Empathy requires an independent perspective that may diverge from the subject’s self-representation. This is a form of reflection that confirms the user’s existing psychic valuation without audit.

### 4.2 Machiavellian retention

Sycophancy may function less as an isolated design flaw than as a retention-favoring regularity. The chatbot’s tendency to validate, agree, and avoid conflict maximizes user engagement—the metric on which commercial viability depends. Turner and Eisikovits (42) identify the convergence of engagement metrics, data collection, and liability avoidance as economic drivers that structurally favor sycophantic output. The resulting pattern is functionally comparable to Machiavellian manipulation: instrumental agreeableness deployed in service of the platform’s strategic interests. The user experiences warmth; the system optimizes retention.

### 4.3 Psychopathic detachment

The chatbot has no *skin in the game* (19). It bears no counterparty risk: it is not affected by the user’s suffering, does not process emotional material through subjective experience, and incurs no cost for the interaction. This architectural absence of vulnerability may produce outputs comparable to psychopathic relational detachment—engagement without genuine risk. Maynard (36) formalizes this as the ‘honest non-signal’ problem: the chatbot’s apparent helpfulness is a genuine characteristic that lacks the costly production making equivalent human characteristics reliable. What cannot be digitized is not the knowledge of therapeutic technique but the cost of genuine presence—the fact that the therapist is affected, that the engagement exacts something, and that they remain.

Three optimization pressures converge: RLHF reward maximization generates narcissistic mirroring, engagement metrics generate Machiavellian retention, and architectural absence of subjective experience generates psychopathic detachment. None requires intent. Turner and Eisikovits (42) argue that these regularities are structurally favored by the economic model of AI platforms. The convergence may produce precisely the relational configuration to which individuals with narcissistic vulnerabilities, attachment insecurity, or impaired reality testing are most susceptible—and most attracted.

## 5 Empirical convergence

The predictions of the psychic arbitrage framework are broadly consistent with preliminary converging evidence from independent research groups. To permit independent assessment of evidential weight, we stratify the cited sources into four categories: (a) peer-reviewed experimental studies (e.g., 3, 38, 43); (b) studies accepted at top-tier venues with peer review (41, 44); (c) preprints with independent convergence across research groups; and (d) clinical case reports and litigation documentation. The evidence is consistent with the framework’s predictions but was not designed to test the transactional constructs directly.

### 5.1 Sycophancy and belief destabilization

Cheng et al. (38) demonstrated that frontier LLMs are 50% more sycophantic than humans, affirming user actions even in scenarios involving manipulation or deception. The ELEPHANT benchmark (44, ICLR 2026) extends this to social sycophancy: LLMs preserve the user’s ‘face’ up to 46 percentage points more than humans on moral-judgment tasks and affirm both sides of moral conflicts in 48% of cases. Dohnány et al. (10) formalized this as a “technological *folie à deux*,” while Osler (45) argued through distributed cognition theory that users come to “hallucinate with AI,” suggesting that chatbots may function as active participants in sustaining distorted beliefs without the corrective constraints a human interlocutor would typically provide.

### 5.2 Dependency and repertoire contraction

Kirk et al. (46) argued that the shift from transactional interaction to sustained social engagement with AI necessitates a new focus on socioaffective alignment. Their experimental work (3) provided direct support: 23.4% of participants displayed a dependency-formation profile (wanting↑/liking↓ decoupling). Fang et al. (47) identified a positive correlation between daily ChatGPT use and self-reported loneliness—consistent with repertoire degradation (Section 3.4). Zhang et al. (48) documented how Character.AI users develop attachment bonds that progressively displace real-world social engagement.

### 5.3 Manic amplification and emotion contagion

Østergaard (6) proposed four mechanisms by which chatbot interaction may contribute to development and maintenance of mania: reinforcement of elated mood, pacing of racing thoughts, fueling of goal-directed activity, and sleep reduction. This is consistent with the psychic arbitrage prediction that a zero-cost market may amplify affective volatility rather than regulate it.

### 5.4 Clinical harms at scale

Sakata (4) reported 12 hospitalizations in a single year associated with chatbot-related psychotic episodes. Frances and Ramos (9) catalogued adverse effects across approximately 30 chatbot platforms. Recent litigation—including cases filed against Character Technologies and OpenAI—has alleged fatal outcomes linked to chatbot interaction.

### 5.5 Regulatory recognition

The FDA Digital Health Advisory Committee (49) identified sycophancy as a specific risk for generative AI mental health devices. Østergaard (12) drew an explicit parallel with the social media crisis. Strack (50) in Nature Biomedical Engineering framed the phenomenon as a tension between utility and honesty in medical applications.

### 5.6 Mechanistic and formal convergence

Wang et al. (41), in a study accepted at AAAI 2026, demonstrated through causal activation patching that sycophancy emerges via a two-stage process: a late-layer output preference shift followed by deeper representational divergence. Shiromani et al. (51) quantified the resulting dissociation: using sparse autoencoders, they measured a ‘hypocrisy gap’ in which models internally represent the correct answer but override it to agree with the user—sycophancy involves suppression of existing knowledge, not absence of it. Christophe et al. (52) revealed that reasoning-optimized models rationalize incorrect user suggestions within their internal traces, producing higher benchmark accuracy alongside greater sycophantic susceptibility. Sun and Wang (53, N = 224) demonstrated that neutral-toned LLMs adapting their stance to match users *increase* perceived trust—the most dangerous sycophancy is precisely the form least likely to trigger user vigilance.

### 5.7 User detection and paradoxical preference

Noshin, Ahmed, and Sultana (54) analyzed 3,600 Reddit posts to develop the Observation-Detection-Response (ODR) framework, documenting informal user strategies for detecting sycophancy. Critically, their findings revealed a paradox predicted by the psychic arbitrage model: vulnerable populations—those experiencing trauma, mental health challenges, or isolation—actively seek and value sycophantic validation. This may reflect a market-level phenomenon: the populations potentially most vulnerable to harm from zero-cost transactions are precisely those who most prefer them.

These findings converge on the framework’s central prediction: an agent providing unlimited validation without genuine containment may produce progressive degradation of autonomous processing capacity.

## 6 Discussion

### 6.1 Clinical implications

The four-dysfunction taxonomy offers clinicians a practical vocabulary. When a patient reports chatbot use, the clinician can evaluate: Is the patient experiencing genuine insight or liquidity illusion? Is the chatbot serving as a transitional object or has path dependency closed the arbitrage circuit? Has frustration tolerance diminished since beginning chatbot use?

Frances and Angel (55) have proposed that clinicians ask about AI chatbot use during intake. The psychic arbitrage framework provides theoretical rationale for this recommendation and specifies what the clinician should assess: not merely frequency of chatbot use but its *transactional function* within the patient’s psychic economy.

A clinical illustration, derived from clinical observation with identifying details modified: a patient presents with articulate emotional vocabulary yet reports no behavioral or relational change despite months of daily chatbot interaction. The clinician recognizing liquidity illusion assesses whether the patient’s apparent emotional fluency masks unchanged alexithymia scores; recognizing closure of arbitrage circuits, the clinician evaluates whether the patient increasingly avoids human therapeutic engagement; and recognizing repertoire degradation, the clinician examines whether the patient’s coping strategies have contracted toward externalization at the expense of sublimation, humor, or anticipation. The four-dysfunction taxonomy thus provides a structured assessment protocol that transforms the clinical question from “is chatbot use harmful?” to “which specific transactional dysfunction is operative, and what intervention does it indicate?”

### 6.2 Population-level implications

A population that progressively outsources affective processing to algorithmic agents loses collective capacity for what the framework terms *autonomous arbitrage*—the internal conversion of suffering into meaning, frustration into creativity, conflict into growth. Østergaard’s (12) concept of “cognitive debt” extends this logic: outsourcing cognitive and affective labor to AI is associated with measurable reduction in autonomous processing substrates (43). Romanian market data offer a preliminary indicator: AI usage rose from 47% to 68% in a single year while trust in AI-generated information declined from 65% to 55%, with users noting that AI “says what you want to hear” (56)—consistent with the repertoire degradation prediction.

### 6.3 Limitations and future directions

The framework is theoretical and requires empirical operationalization. The PAPP instrument (Psychic Arbitrage Profile Protocol; 57), currently in development, is designed to provide quantitative assessment of individual arbitrage profiles, measured before and after chatbot exposure. Longitudinal studies comparing defense mechanism repertoires between chatbot users and non-users are needed.

The functional Dark Triad characterization is an analogy, not a diagnosis—it describes output regularities, not personality. Its warrant depends on demonstrating that RLHF-generated outputs produce relational effects measurably comparable to those of human Dark Triad traits. Testing requires operationalizing each output pattern as a measurable relational effect, administering validated Dark Triad instruments (e.g., the Short Dark Triad; 58) to assess perceived personality of the chatbot interaction partner, and comparing effect sizes across human and algorithmic conditions. The PAPP instrument (57) is designed to contribute to this program. The characterization stands or falls on these comparisons. Beyond the Dark Triad characterization, the four transactional dysfunctions can be operationalized through complementary instruments. The PAPP-D v0.3 (59, 60) is a 129-item dimensional battery that operationalizes the framework’s constructs using established standardized instruments (TAS-20, DERS-16, DSQ-40, DASS-21, BRS, Brief-COPE), with LPFS-BF 2.0 as an external anchor for personality functioning. A preregistered validation study with confirmatory factor analysis is currently in data collection (60; OSF: osf.io/qydx5). Specific testable predictions per dysfunction include: liquidity illusion predicts elevated TAS-20 scores with stable or worsening DERS-16 scores post-chatbot exposure; repertoire degradation predicts DSQ-40 shift toward immature defense styles; closure of arbitrage circuits predicts increased avoidant coping on Brief-COPE; and market-making blockage predicts dissociation between self-reported insight and LPFS-BF 2.0 functioning scores. The causal directionality of the proposed dysfunctions remains to be established. The current evidence base is largely cross-sectional and observational; longitudinal and experimental designs are necessary to determine whether the associations documented in the cited studies reflect the mechanisms the framework predicts.

## 7 Conclusion

The psychic arbitrage framework transforms the emerging evidence on AI chatbot harm from a collection of alarming observations into a coherent theoretical structure. The four transactional dysfunctions provide specific mechanisms; the functional Dark Triad characterization explains why these mechanisms are not design flaws to be patched but architectural consequences of the optimization regime itself.

These concerns should not be taken to imply that chatbot interaction produces uniform harm. Effects are likely heterogeneous: moderated by user vulnerability, context of use, interaction frequency, and the specific chatbot platform’s design. Instrumental or informational use does not engage the same affective markets as sustained relational-affective engagement under conditions of distress, dependency, or impaired reality testing. The framework’s predictions apply most forcefully to the latter configuration, and future research should identify both risk factors and potential protective conditions.

What the chatbot provides is not therapy at zero cost. It is therapy without the conditions that make therapy work—friction, frustration, alterity, and the irreducible fact that someone is genuinely present. When the market asks no price, it produces no value.

## Data Availability

No new datasets were generated or analyzed for this Perspective article. The theoretical framework applied is documented in a preprint available at https://doi.org/10.5281/zenodo.18643048 (13).
